# Profile Distributions of Potentially Toxic Metal(loid)s in Soils of the Middle Odra Floodplain (SW Poland)

**DOI:** 10.3390/ijerph20054196

**Published:** 2023-02-26

**Authors:** Dorota Kawałko, Anna Karczewska

**Affiliations:** Institute of Soil Science, Plant Nutrition and Environmental Protection, Wrocław University of Environmental and Life Sciences, ul. Grunwaldzka 53, 50-357 Wrocław, Poland

**Keywords:** alluvial soils, arsenic, contamination, farmlands, heavy metals, inter-embankment zone, local geochemical background, risk assessment

## Abstract

Floodplain soils are often contaminated with potentially toxic elements of geogenic and anthropogenic origin. This also applies to a valley of the Odra river, which in its upper reach flows through areas of historical and contemporary mining and heavy industry. This study examined the distribution of typically anthropogenic metal(loid)s, i.e., Pb, Zn, Cu, As and Cd, and geogenic metals, i.e., Mn and Fe, in soil profiles of the middle Odra valley, and analyzed factors that determine their concentrations. Thirteen soil profiles, located inter the embankment area and outside the embankments, were examined. Most of profiles indicated stratification typical for alluvial soils. Topsoil layers in the inter-embankment zone showed considerable enrichment in Pb, Zn and Cd, and to a lesser extent in Cu and As. Low soil pH is an important factor of environmental risk; therefore, acidic soils definitely require liming. The soils located out of embankments did not show any considerable enrichment in the elements examined. Based on significant correlations between the concentrations of metal(loid)s in deep soil layers and soil texture, the values of local geochemical background were derived. Outliers, particularly in the case of As, were explained by possible redistribution under reducing conditions.

## 1. Introduction

Floodplain soils are often contaminated with potentially toxic elements, particularly when they adversely affected by effluents from mining or industrial areas. Possible sources of metals include discharges of insufficiently treated wastewater, seepage from poorly protected mine dumps and historical landfills, as well as diffuse input from the catchments [1,2,3,4,5,6]. The contaminants can be transported by the river, sometimes over long distances, in the forms of both dissolved and suspended matter. Metals and metalloids, however, tend to precipitate relatively soon and enrich the bottom sediments, in particular in the close vicinity of their source. Enriched sediments can also be a potential secondary source of contamination. Their transport over longer distances, often associated with flood events, can cause the accumulation of water-borne contaminants in floodplain soils [7,8,9,10]. Such enrichment occurs mainly in the surface levels of alluvial soils, up to a certain depth. However, metal(loid)s can be further subject to remobilization. Soil enrichment in various elements at longer distances from the source of contamination, the relationships among the elements and their profile distributions in soils can provide information on their environmental behavior.

**Figure 1:**
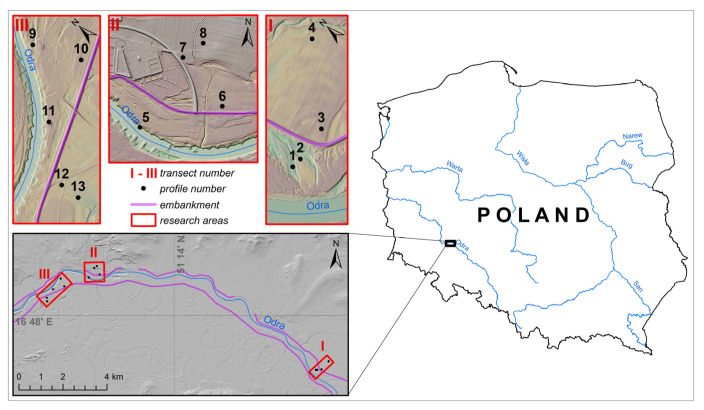
Study area and situation of soil profiles.

These issues also apply to the floodplains of the Odra river, the second largest river of Poland (Figure 1), which, in its upper reach, flows through Upper Silesia, the region of historical and contemporary coal and Zn-Pb ore mining and heavy industry [11,12,13]. In addition, tributaries of the upper Odra river are also heavily polluted [14,15,16]. The amounts of underground water from ore mines, as well as from coal mines, discharged into the Odra river reached their maximum values in the second half of the 20th century [17]. Several authors confirmed that the sediments in the upper Odra reach, upstream of Wrocław city, are strongly enriched in metals. Many studies on the redistribution of metals in river sediments have been carried out in this part of the Odra valley. Some of them focused on the displacement of sediments during the river regulation and modification of the embankment system. The groyne fields filled with highly contaminated sediments were then dissected by new bank lines, and their material was partially keyed into the floodplains of the upper Odra reach [13,18].

In addition to the upper part of the Odra river valley, studies on river sediments were also carried out in the lower reaches of the river [19,20,21], downstream of Głogów, where water from the largest European center of copper ore mining and processing is discharged [22]. They confirmed the enrichment of sediments in heavy metals, in particular Cu and Pb, in that part of the river system.

Unlike the upper and lower reaches of the Odra river, its middle reach has not been so extensively examined in terms of the accumulation of metal(loid)s in sediments and floodplain soils. Related data are scarce, mainly of a screening nature [18,21,22,23,24,25]. This part of the river is much less influenced by contaminants released from Upper Silesia, but water discharge from the city may be an additional source of contamination. To this day, no soil-science-oriented studies have been carried out on the distribution of metals in deep profiles of alluvial soils in the middle Odra valley. It should be emphasized that the profile distribution of elements can be significantly influenced by soil properties and pedological processes. Some metal(loid)s, for instance Cd and Zn, considered mobile and susceptible to remobilization, may migrate downward the soil profile or may be transported with water over greater distances [26,27,28]. Other metal(loid)s, particularly Mn and As, as well as Fe, are susceptible to reductive dissolution and mobilization under reducing conditions [29,30,31,32]. On the contrary, Pb is considered relatively immobile [33,34]. Therefore, the relationships between the concentrations of various elements of the same origin in soils may differ along the river course.

The present study aimed to: (1) examine the extent to which the soils in the Odra valley, in its middle course, are enriched in toxic elements; (2) describe their profile distribution, which will allow us to discuss their origin and fate; (3) check whether there are considerable differences between the inter-embankment zone and the sites out of embankments; (4) attempt to determine local geochemical background of elements based on their concentrations in deep soil layers differing in texture. Additionally, the conditions required for the safe use of contaminated floodplain soils for agricultural purposes are discussed.

## 2. Materials and Methods

### 2.1. Research Area and Location of Soil Profiles

The research was carried out in the Odra river valley, in its middle course, downstream from Wrocław (Figure 1), along the length of 15 km. The Pleistocene terraces, shaped by fluvioglacial water of Weichselian glaciation, are mainly built of sands and gravels, while the Holocene terraces contain fine-textured interbeddings. The top layers of floodplains are typically built of loams or silts [35,36,37]. Channeling of the river and the construction of embankments at the beginning of the 20th century effectively protected the zones out of embankment from floods and additionally led to lowering of the groundwater table [18,38]. These changes created favorable conditions for the agricultural use of soils and the replacement of riparian forests by farmlands and pastures by plowed fields [39]. The inter-embankment areas are also used for agricultural purposes, mostly as meadows or pastures.

Thirteen soil profiles examined in this study were situated in three transects (Figure 1) and included six profiles in the inter-embankment zone and seven profiles outside of embankments. All the sites represented agricultural lands: pastures, meadows and arable fields.

### 2.2. Soil Sampling and Analysis

Soil pits were dug to a depth of 1.5 m or to the level of groundwater. Profile No. 1 was relatively shallow, with a silty clay loam texture and the groundwater depth of 45 cm. All soil profiles were classified and described according to the FAO-WRB [40]. The samples were collected from all morphologically distinguishable horizons and layers. Basic soil properties were determined in representative aliquots of collected samples. Soil texture was determined using a combined sieve and hydrometer method [41]. Chemical analyses were carried out with the methods commonly used in soil science [42], after soil was ground to a fine powder. Soil pH was measured potentiometrically in a suspension in 1 M KCl (1:2.5; *v*/*v*). A dry combustion method (Vario MacroCube, Elementar) was used for the determination of organic carbon (Corg). Total concentrations of Pb, Zn, Cu, As, Cd, Mn and Fe were determined, after soil digestion with aqua regia (concentrated HCl + HNO_3_, 3 + 1) in a microwave system, according to ISO 11466 [43]. The concentrations of elements in the digests were measured via ICP-AES (iCAP 7400, Thermo Fisher Scientific). All analyses were made in triplicates. The accuracy of the results was checked with two reference materials: CNS 392 and CRM 027, supplied by Sigma-Aldrich, certified for aqua regia extracted elements. The recoveries of elements in CRMs ranged 82–124% for Cd and 94–109% for all the elements, which we considered satisfactory.

### 2.3. Data Analysis

Basic descriptive statistics was applied to examine the distributions of data in all the samples as well as in the subsets defined as: inter-embankment zone (In), out of embankment zone (Out), topsoil A horizons (Top) and deep soil layers (>60 cm). The first comparison of the results between the groups was based on the medians, and further, the data were subject to analysis of variance (ANOVA) followed by Tukey’s test to assess the significance of differences between the means at *p* < 0.05. Shapiro–Wilk’s normality test (*p* < 0.05) was applied to check the normality of data distribution. Since the distributions of element concentrations in the entire sets of data were not normal and showed high skewness, the relationships between the concentrations of elements and the parameters of soil properties were examined using a non-parametrical test, in which Spearman correlation coefficients were calculated at *p* < 0.001. After applying log-transformation, the distributions became close to normal; therefore, a principal component analysis was used in order to illustrate the associations between the variables, as often applied [44,45,46,47]. Only those principal components that contributed to a total variance more than 10% were taken into account. For a subgroup of deeper, uncontaminated horizons, where the data showed distributions close to normal, the equations of simple linear regression were derived to describe key relationships between the concentrations of elements and the parameters of basic soil properties. Statistical analyses were performed using Statistica 13.0 software (TIBCO, Palo Alto, CA, USA).

## 3. Results and Discussion

### 3.1. Basic Soil Properties

All soils showed a stratification typical for alluvial soils [48,49,50]. Soil texture showed a broad spectrum from sands, through loams and silts to clays. The clay texture was found in the deep layers of profile 13, out of the embankment, while the presence of layers built of sand was a typical feature for deeper parts of soil profiles both in the inter-embankment and out of the embankment. The top layer of soils in the inter-embankment zone was typically, except for profile 1, developed of silt–loamy material. The depth of this layer was different in various soil profiles, with a maximum of 100 cm in profile 9. Table 1 presents the aggregate data on soil properties in all collected soil samples, as well as in the subgroups: inter-embankment zone (In), out of embankment (Out), topsoils (Top) and deeper layers (>60 cm).

The median content of silt fraction in the group of samples collected from the inter-embankment zone was 47%, which was much higher than that in the samples from the profiles situated out of embankments (18%), most of which had sandy textures (Table S1). The values of soil pH were in a very broad range: 3.40–7.37. Strongly acidic soils (with pH < 4.0) were present in both zones: in profiles 1 and 2 in the inter-embankment zone as well as in profile 13 out of the embankment. Soil CEC values differed widely, ranging from 0.4 to 34.3 mmol(+)/kg, and were clearly dependent on the content of Corg in topsoil samples and on the content of clay fraction in deeper layers.

### 3.2. Metal(loid)s in Soil Profiles

The concentrations of metal(loid)s in soil samples differed widely (Table 2). Particularly high concentrations of typically anthropogenic elements, i.e., Pb, Zn, Cu, As and Cd, occurred in the inter-embankment zone in the surface soil layers (Top). This group of samples was additionally the richest in Corg (Table 1), with a median of 41.4 g/kg Corg and the highest median CEC of 17 cmol(+)/kg.

**Figure 2:**
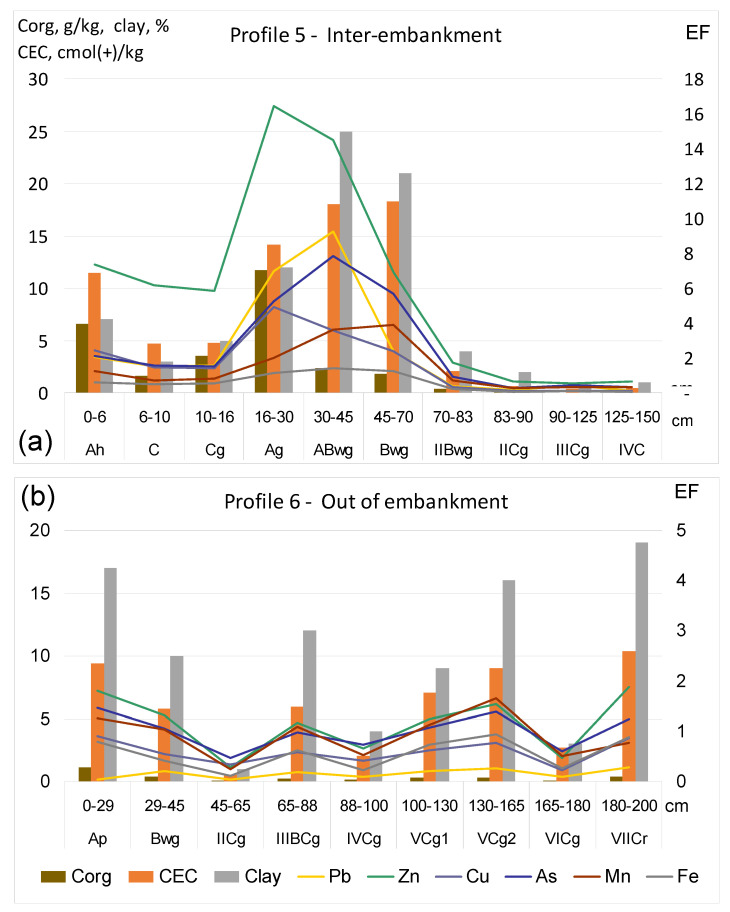
Distribution of metal(loid)s, against the background of crucial parameters of soil properties, in profiles 5 (**a**) and 6 (**b**), representative of the inter-embankment and out of embankment zones, respectively, in transect No. II. EF—enrichment factor calculated in relation to geochemical background; details are provided in the text.

**Figure 3:**
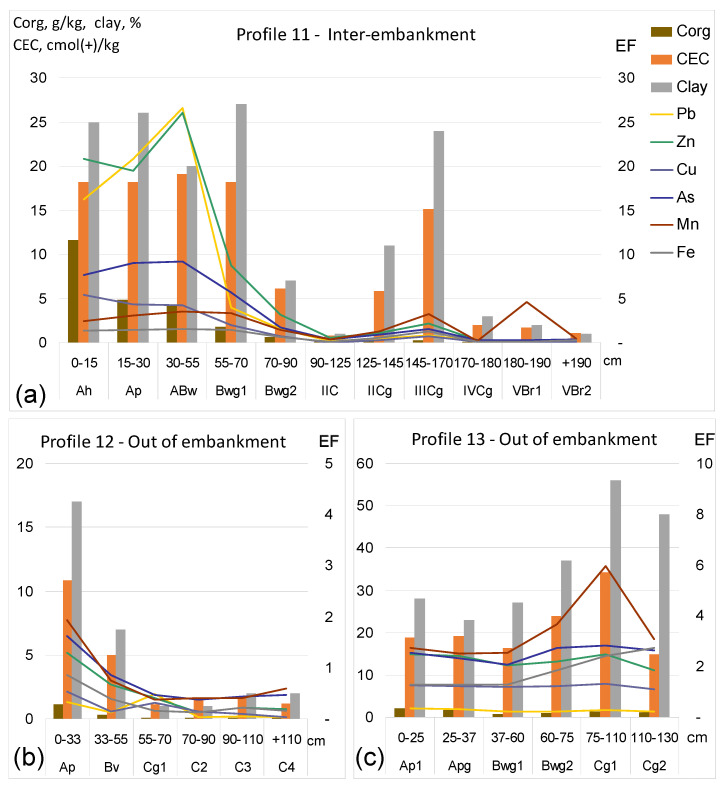
Distribution of metal(loid)s, against the background of crucial parameters of soil properties, in profiles 11 (**a**) in inter-embankment zone, 12 (**b**) and 13 (**c**) out of embankments, in transect No. III. Deeper soil layers in profile 12 had the light texture of sand, while those in profile 13 were much heavier, i.e., clay loam and clay. EF—enrichment factor calculated in relation to geochemical background; details are provided in the text.

Illustrations of profile distributions of all metal(loid)s and crucial soil properties in the profiles representative of inter-embankment and out of embankment-zones, in transects II and III, are shown in Figure 2a,b and Figure 3a–c. Due to the fact that the concentrations of individual elements fell in different orders of values, the graphs illustrate the enrichment factors (EFs) instead of absolute concentrations of metal(loid)s. The values of EF were calculated in relation to concentrations commonly accepted as the geochemical background (Table 3), i.e., the median values for European subsoil, according to FOREGS [51,52].

The graphs clearly show that the patterns of profile concentrations of metal(loid)s in soils of transect II (Figure 2a,b) mimic those of CEC values, which are determined mainly by the content of Corg in topsoil and by clay in deeper layers. A particular morphological feature of profile 5, important for the distribution of soil properties and concentrations of metal(loid)s, is the presence of buried, organic-matter-rich Ag (16–30 cm) and ABwg (30–45 cm) horizons, of silty–loam texture, covered by sandy and sand–loamy layers, in which a histic horizon Ah (0–6 cm) has already developed.

The maximum concentrations of typically anthropogenic elements, i.e., Pb, Zn, Cu, As and Cd, in this profile were found in the Ag buried horizon, although in the topsoil layers, they also significantly exceeded the geochemical background. The EF values for these layers were much above 1, and for Zn, they reached a value of 16. Undoubtedly, sediments that build the topsoil layers in this part of the embankment zone were deposited in a relatively recent time, probably during hydrotechnical works reported by Ciszewski and Grygar [9].

Unlike profile 5, the other soil profiles of the inter-embankment zone did not show any disorders in the layout of horizons typical for alluvial soils. The surface soil layers in all the profiles situated in the inter-embankment zone contained relatively high concentrations of metals. Profile 11 turned out to be most contaminated, with very high concentrations of Pb, Zn, Cu, As and Cd in the Ah, ABw and Ap horizons, down to the depth of 55 cm (Figure 3a). Those concentrations exceeded the permissible levels for agricultural soils, defined in Polish law [53] and discussed further in Section 3.5.

In contrast to the soils in the inter-embankment zone, those situated out of the embankment showed no significant enrichment in metal(loid)s in either the surface or deeper horizons. The exception was As in profile 8, with clearly elevated concentrations in deeper soil layers, with a maximum of 24.5 mg/kg in the Cg3 horizon at a depth of 60–80 cm. This horizon was also very rich in aqua regia-extractable Fe (>5%). This concurrence of those two elements is not surprising, confirming a well-known affinity of As to Fe hydroxides [55,56]. Though, this may indicate the importance of ancient hydromorphic processes on the distribution of As in soils, not only in the inter-embankment but also outside the embankment. The source of soil enrichment in As is unknown, but Szerszeń et al. [57] also reported relatively high As concentrations in the deeper layers of alluvial soils, rich in Fe, in the further course of the Odra river, regardless of soil texture.

**Figure 4:**
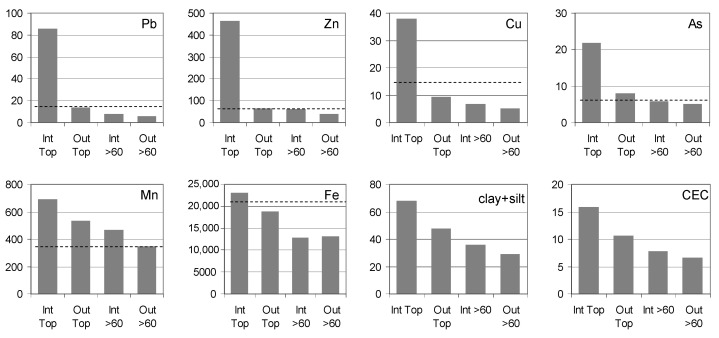
Mean concentrations of metal(loid)s: Pb, Zn, Cu, As, Mn and Fe in soils (mg/kg), as well as the mean values of clay + silt content (%) and CEC (cmol(+)/kg). Median values reported by FOREGS (Table 3) for European subsoil are shown with dashed lines.

Figure 4 shows the mean concentrations of metal(loid)s in the samples that belong to four distinguished subgroups, i.e., the surface humus layers (Top) and deeper, mineral layers (>60 cm), collected from the profiles situated in the inter-embankment zone (Int) and out of the embankment (Out). Cadmium was not included in the graph because its concentrations in most samples, particularly in sandy ones, were extremely low, i.e., below the ICP-AES limit of quantification (0.14 mg/kg). Therefore, for Cd, it was not possible to quantitatively determine the means, and only the median values were provided (Table 1). The mean concentrations of other metal(loid)s were compared with the reference values for European subsoils, according to FOREGS [51,52].

The highest concentrations of all metal(loid)s definitely occurred in the subgroup “topsoil in the inter-embankment” (Figure 4). In the case of Pb, Zn and Cu, the maximum concentrations were several times higher compared to the other subgroups and to the European reference values.

### 3.3. Factors That Determine Soil Concentrations of Metal(loid)s

The above-presented observations concerning the patterns of profile distribution of elements in soil profiles were confirmed by statistical analysis. Calculated Spearman correlation coefficients (Table 4) indicated strong relationships between the concentrations of elements and soil parameters.

**Figure 5:**
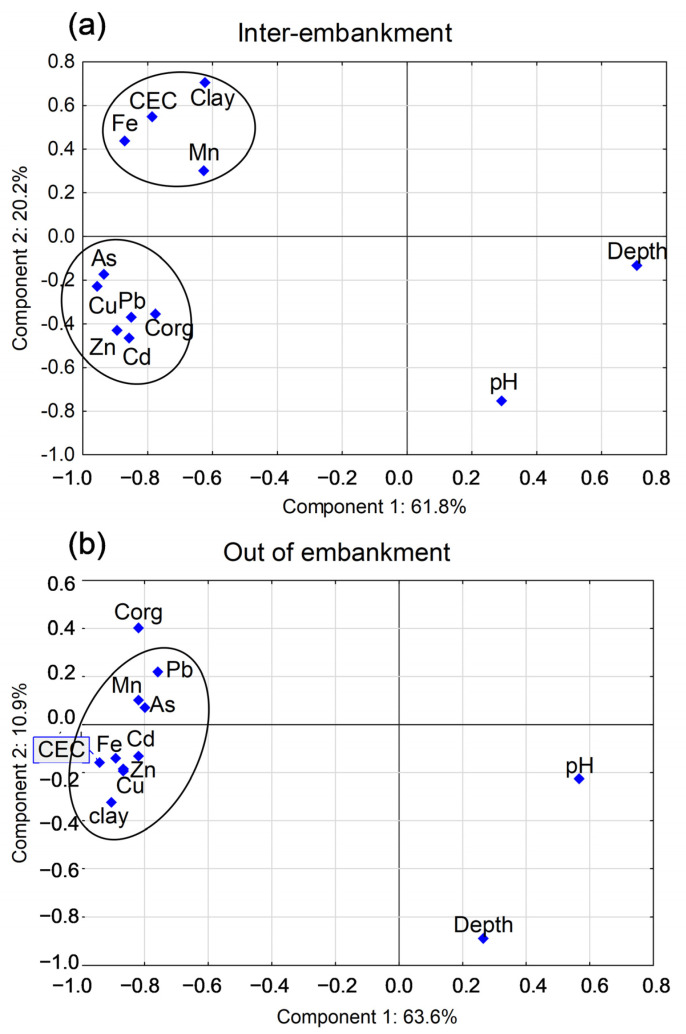
The results of PCA analysis carried out for: (**a**) inter-embankment (above) and (**b**) out-of-embankment (below) soil samples, separately.

As some of those parameters are interrelated, for instance soil texture and CEC, therefore, the PCA analysis was used to illustrate the complex relationships. This analysis was carried out separately for the soils of the inter-embankment and out-of-embankments zones, with log-transformed concentrations of metal(loid)s. The results confirmed strong relationships between the elements originating from anthropogenic sources, i.e., Pb, Zn, Cu, As, and Cd, and their accumulation in inter-embankment soils, mainly in their surface horizons, rich in humus (Figure 5a). The concentrations of lithogenic elements, i.e., Mn and Fe, in the soils of the inter-embankment zone proved to be mainly related to soil clay content and the CEC values. In the case of out-of-embankment soils, a similar analysis showed no clear division of elements into anthropogenic and lithogenic groups (Figure 5b), and CEC turned out to be the main factor that determined the total concentrations of all elements in those soils. Detailed data on related correlation coefficients are given in Table S2.

The anthropogenic enrichment of surface soil layers in the inter-embankment zone is therefore beyond doubt. It is worth asking, however, whether in the deeper soil horizons, built of older sediments, the relationships between soil properties and concentrations of metal(loid)s differ between the inter- and out-of-embankment zones. Possible differences between them could result from the translocation of elements, their redistribution and secondary enrichment. Therefore, for the deeper (>60 cm) soil layers of both zones, simple correlations between the concentrations of metal(loid)s and crucial soil parameters—clay content, the sum of clay and silt fractions and CEC values—were analyzed. The most significant correlations (Table S3) were obtained for the sum clay + silt. Related graphs are shown in Figure S1. There were single outliers, in particular in profile 11, with a thick layer of silty sediments strongly enriched in metal(loid)s, in which high concentrations of anthropogenic metal(loid)s occurred to the depth of 70 cm. In the case of As, Mn and Fe, there were also some outliers in profiles 8 and 13 (Figure S1) that had particularly heavy clay textures.

### 3.4. Assessment of Local Geochemical Background

Statistically significant linear relationships between soil concentrations of metal(loids) vs. the sum clay + silt in the subsoils (>60 cm) were quite similar in the inter- and out-of-embankment zones (Figure S1). Therefore, based on related linear equations, it was possible to calculate the concentrations of elements that can be considered natural in the alluvial soils of the Odra valley (Table 5).

Taking into account the discussion concerning the most appropriate methodology of determining the geochemical background [58,59,60,61,62], we believe that these values can be applied as local geochemical background values for soils that have different textures.

### 3.5. Assessment of Soil Contamination and Environmental Risk

The risk associated with soil contamination was assessed according to the Polish legal regulations [53,63] which differentiate permissible concentrations of contaminants according to the categories of soil usage. In the case of farmland topsoils (0–25 cm), the permissible concentrations of metal(loid)s depend on soil texture and pH. At deeper layers (>25 cm), they are only related to soil water permeability. The permissible concentrations of metal(loid)s in farmland soils are shown in Table 2 as reference values. In our study, they were exceeded in two soil profiles, No. 5 and 11, located in the inter-embankment zone. In profile 5, excessive concentrations applied to Zn, Cd and As in the Ag horizon (16–30 cm), which fell mainly in the 0–25 cm layer, and to Zn in ABwg horizon (30–45 cm). In profile 11, excessive concentrations of Zn, Cd and As were also present in the top horizon Ah (0–15 cm) and Ap (15–30 cm). It should be noted that, in this assessment, the current acidic soil reaction was considered. Although, the efficient liming of those soils would result in their shift from the subgroup 2 to subgroup 3, which would allow the requirements for farmlands to be met.

What will still remain problematic, however, are the concentrations of Zn in deeper soil layers (>25 cm) of two profiles, i.e., in the ABwg horizon of profile 5 (30–45 cm) and the ABw horizon in profile 11, which were as high as 638 and 1145 mg/kg, respectively. Those values exceed the permissible concentrations for low permeability subsoil, i.e., 500 mg/kg. In such a situation, the provisions of Polish law say that the assessment of environmental risk should be carried out in order to make a decision on the need for and method of remediation. A rough analysis of possible adverse environmental effects that could be associated with such high concentrations of Zn in soils, indicated, however, that its partial release due to desorption should not cause any massive pollution of water or any risk to arable crops, provided that topsoils are subject to liming. Therefore, assuming that the soils are limed, the overall environmental risk can be assessed as low.

## 4. Conclusions

The soils located both in the inter-embankment zone and out of the embankment showed the stratification which is a typical feature of floodplain soils. The surface horizons of inter-embankment soils are built of silty–loam layers of various depths, considerably enriched in metals of anthropogenic origin, in particular Pb, Zn and Cd, and to a lesser extent also in Cu and the metalloid As. Those soils that proved to have acidic reactions will require liming to reduce the risk associated with their potential use for agricultural purposes. It should be stressed, however, that this problem is confined to the inter-embankment zone, while the soils situated out of the embankment did not show any significant enrichment with potentially toxic elements. The concentrations of all the elements in deeper soil layers (>60 cm) in both zones showed a strong, statistically proven, linear dependence on the parameters related to soils’ sorption properties, i.e., CEC, clay content and the sum of clay + silt, with the latter relationship being statistically most significant. Based on this, the typical concentrations of elements related to soil texture were assessed, which can be used as the values of geochemical background for soils of the Odra valley. Some outliers that did not fit to these relationships may indicate a redistribution of elements. For example, particular enrichment in As in the layers composed of sediments with very heavy textures, rich in Fe, may have resulted from a historical redistribution of As under reducing conditions. Confirming this hypothesis would require more detailed, targeted research.

## Figures and Tables

**Table 1:** Cumulative data on soil properties in all sampled soil samples (N = 99) and in the subgroups: inter-embankment zone (In) and out of embankment (Out), as well as (within them): the top horizons A enriched in organic matter (Top) and deeper layers (>60 cm). Presented are: Min–Max/Median values.

Parameter	All Samples	Inter-Embankment Zone (In)			Out of Embankment (Out)		
		All	Top Layers ^1^	>60 cm	All	Top Layers ^1^	>60 cm
Number of samples	99	51	15	30	48	10	35
Skeleton, % (gravel and stones)	0–25	0–17	0–17	0–2	0–25	0–21	0–25
	0	0	0	0	0	1	0
Sand ^2^, %	8–99	10–99	10–99	16–98	8–99	14–75	10–99
	61	34	23	70	74	59	89
Silt ^2^, %	<1–66	<1–66	0–64	0–63	<1–65	21–63	0–53
	25	47	56	23	18	27	5
Clay ^2^, %	1–56	1–40	3–31	1–40	1–56	4–28	1–56
	12	16	20	11	8	16	5
Clay + silt ^2^, %	1–92	1–90	13–89	1–88	1–92	25–86	1–92
	39	66	76	26	26	42	11
Corg, g/kg	0.3–118	0.3–118	15.3–118	0.3–17.9	0.5–21.1	3.3–21.1	0.5–13.3
	3.5	7.7	41.4	2.5	2.4	11.4	1.3
pH	3.40–7.37	3.66–6.16	3.73–5.32	3.80–6.16	3.40–7.37	3.42–6.17	3.40–7.37
	5.09	4.85	4.83	4.93	5.30	4.75	5.31
CEC, cmol(+)/kg	0.4–34.3	0.4–25.4	4.7–25.4	0.4–25.0	0.5–34.3	3.0–19.1	0.5–34.3
	7.4	13.6	17.0	6.1	4.9	9.9	2.9

^1^ Top layers—A horizons (having the features of Corg accumulation); ^2^ in fine soil (<2 mm).

**Table 2:** Cumulative data on the concentrations of metal(loid)s in all sampled soil samples (N = 99) and in the subgroups: inter-embankment zone (In) and out of embankment (Out), as well as (within them): the top horizons A enriched in organic matter (Top) and deeper layers (>60 cm). Presented are: Min–Max/Median values.

Parameter	All Samples	Inter-Embankment Zone (In)			Out of Embankment (Out)		
		All	Top Layers	>60 cm	All	Top Layers	>60 cm
Number of samples	99	51	15	30	48	10	35
Pb, mg/kg	1.1–266	1.1–266	22.6–266	1.1–39.7	1.2–20.1	1.5–20.1	1.2–19.6
	9.5	13.1	50.3	6.2	4.7	14.5	4.1
Zn, mg/kg	5.1–1145	5.4–1145	105–1145	5.4–385	5.1–138	25.0–110	5.1–138
	58	86	324	45	29	60	27
Cu, mg/kg	0.2–74.7	0.4–74.7	16.5–74.7	0.4–27.4	0.2–22.1	3.6–17.6	0.2–22.1
	7.4	11.9	34.1	5.0	4.2	7.4	2.6
As, mg/kg	1.0–45.8	1.2–45.8	7.6–45.8	1.2–28.5	1.0–24.5	2.5–13.9	1.0–24.5
	6.2	8.6	16.1	4.4	3.6	7.7	3.0
Cd, mg/kg	<0.14–3.57	<0.14–3.57	0.40–3.57	<0.14–1.04	<0.14–1.36	0.40–0.86	<0.14–1.36
	0.40	0.56	1.57	0.40	0.40	0.40	0.40
Mn, mg/kg	15–2010	31–1560	238–1220	31–1560	15–2010	220–916	15–2010
	383	569	678	366	253	465	179
Fe, g/kg	1.22–57.5	12.1–32.3	10.7–32.3	12.2–31.2	1.67–57.5	3.60–47.4	1.67–57.5
	13.3	19.5	23.7	10.9	9.3	17.3	7.34

**Table 3:** Reference concentrations of metal(loid)s in soils according to various sources.

Element	Geochemical Background						Permissible Values				
	Agricultural Soils Ap Horizon ^3^		According to FOREGS ^1^				According to Polish Law ^2^				
			Topsoil		Subsoil		Topsoil, Subgroups			Subsoil, with Permeability	
	Q25	Median	Min.	Median	Min.	Median	1	2	3	H	L
Pb	6.7	9.7	<3	15	<3	10	100	250	500	100	300
Zn	20	30	4	48	5	44	300	500	1000	300	500
Cu	5.4	9.4	1	12	<1	13.9	100	150	300	100	200
As	1.2	2.3	<5	6.0	<5	5.0	10	20	50	20	50
Cd	0.08	0.12	<0.01	0.145	<0.01	0.09	2	3	5	3	5
Mn	154	280	<10	382	<10	337	Not determined				
Fe	No data		700	19,600	700	21,100					

^1^ FOREGS [51,52]; ^2^ Polish Journal of Laws [53]; subgroups for farmland topsoil (0–25 cm) distinguished according to fraction < 0.02 mm, pH and Corg; permissible values for subsoil: H—with high permeability (>10^−7^ m/s) and low permeability (≤10^−7^ m/s); ^3^ agricultural soils of North Europe, GEMAS [54].

**Table 4:** Single Spearman correlation coefficients between the main parameters of soil properties and concentrations of metal(loid)s. All the correlations proved significant at the level of 99.9% (*p* < 0.001). Particularly high values of coefficient (>0.840) have been highlighted in bold.

Element	Clay	Clay + Silt	Corg	pH	CEC	Pb	Zn	Cu	As	Cd	Mn
Pb	0.685	0.775	**0.899**	−0.451	0.804	x					
Zn	0.754	0.819	**0.897**	−0.366	**0.847**	**0.898**	x				
Cu	0.761	**0.843**	**0.895**	−0.412	**0.852**	**0.907**	**0.938**	x			
As	0.775	**0.848**	**0.876**	−0.482	**0.865**	**0.846**	**0.851**	**0.853**	x		
Cd	0.554	0.615	0.759	−0.372	0.671	0.705	0.727	0.726	0.742	x	
Mn	0.647	0.734	0.690	−0.471	0.721	0.652	0.664	0.656	0.832	0.573	x
Fe	**0.913**	**0.939**	0.813	−0.526	**0.943**	0.763	0.808	0.815	**0.916**	0.675	0.798

**Table 5:** Assessed values of geochemical background in alluvial soils of the middle Odra valley as related to soil texture (the sum of clay + silt/or sand fractions).

Clay + Silt	Sand	Pb	Zn	Cu	As	Mn	Fe
%	%	mg/kg	mg/kg	mg/kg	mg/kg	mg/kg	g/kg
5	95	3.0	19	1.5	2.2	185	3.8
10	90	3.7	25	2.3	2.8	225	5.5
20	80	5.1	36	3.9	3.9	304	8.9
30	70	6.5	47	5.6	5.1	384	12.3
40	60	7.9	58	7.2	6.2	463	15.7
50	50	9.4	69	8.9	7.4	543	19.1
60	40	10.8	81	10.5	8.5	622	22.6
70	30	12.2	92	12.1	9.7	702	26.0
80	20	13.6	103	13.8	10.8	781	29.4
90	10	15.0	114	15.4	12.0	861	32.8

## Data Availability

The data that support the findings of this study are available from the authors upon request.

## Supplementary Materials

The following supporting information can be downloaded at: https://www.mdpi.com/article/10.3390/ijerph20054196/s1, Table S1: The numbers of soil samples that represented various textural groups and the ranges of pH in all soil samples (N = 99) and in the subgroups: inter-embankment zone and out of embankment; Table S2: The correlations of particular parameters with principal components determined in the PCA analysis; Table S3: Single Pearson correlation coefficients calculated for deep soil samples (>60 cm); Figure S1: The relationships between soil concentrations of metal(loid)s and the sum of clay + silt fraction in the subsoil (>60 cm).
