# Supplementary material for: Profile Distributions of Potentially Toxic Metal(loid)s in Soils of the Middle Odra Floodplain (SW Poland)
Source: Int J Environ Res Public Health. 2023 Feb 26;20(5):4196. doi: 10.3390/ijerph20054196 (PMC10001738; doi:10.3390/ijerph20054196)
Supplement: Supplementary file 1 [file ijerph-20-04196-s001.zip › ijerph-2224936-supplementary.pdf]

Table S1. The numbers of soil samples that represented various textural groups and the ranges of pH in all soil samples (N = 99) and in the subgroups: inter-embankment zone and out of embankment

Samples	The number of samples that represented soil texture of				The number of samples that had pH in the range			
	sand	silt	loam	clay	<4.5	4.5-5.5	5.5-6.5	>6.5
All samples	40	24	32	3	17	56	25	1
Inter-embankment	17	22	12	0	12	30	9	0
Out of embankment	23	2	20	3	5	26	16	1

Table S2. The correlations of particular parameters with principal components determined in the PCA analysis. The correlations significant at $p < 0.001$ are marked with asterisks.

Parameter	Inter-embankment		Out of embankment		
	Component 1	Component 2	Component 1	Component 2	Component 3
Clay	-0.751*	0.585	-0.925*	0.293	0.001
clay+silt	-0.878*	0.443	-0.939*	0.169	-0.132
Corg	-0.697	-0.476	-0.799*	-0.428	-0.169
CEC	-0.875*	0.408	-0.955*	0.133	-0.064
pH	0.423	-0.666	0.548	0.316	-0.513
Pb	-0.776*	-0.522	-0.753*	-0.210	-0.330
Zn	-0.799*	-0.582	-0.884*	0.200	-0.329
Cu	-0.888*	-0.427	-0.886*	0.199	-0.262
As	-0.897*	-0.334	-0.791*	-0.132	0.169
Cd	-0.773*	-0.597	-0.806*	0.021	0.443
Mn	-0.675	0.183	-0.794*	-0.212	0.381
Fe	-0.914*	0.357	-0.888*	0.075	0.166
Depth	0.715*	-0.018	0.238	0.855*	0.304
Contribution	0.625	0.216	0.672	0.097	0.078
Total contribution, %	84.1%			84.7%	

Table S3. Single Pearson correlation coefficients calculated for deep soil samples (>60 cm, N=51). The correlations significant at $p < 0.001$ are marked with asterisks.

Parameter	clay	Corg	CEC	pH	Pb	Zn	Cu	As	Cd	Mn
clay	x	0,856*	0,959*	-0,445	0,826*	0,866*	0,860*	0,732*	0,760*	0,577*
clay+silt	0,977*	0,880*	0,966*	-0,492*	0,890*	0,907*	0,910*	0,738*	0,717*	0,601*
Corg		x	0,875*	-0,565*	0,849*	0,774*	0,799*	0,683*	0,819*	0,654*
CEC			x	-0,462*	0,849*	0,876*	0,865*	0,719*	0,694*	0,631*
pH				x	-0,426	-0,244	-0,408	-0,486*	-0,534*	-0,543*
Pb					x	0,929*	0,949*	0,651*	0,540*	0,601*
Zn						x	0,920*	0,622*	0,469*	0,457*
Cu							x	0,612*	0,538*	0,618*

As	x	0,565*	0,567*
Cd		x	0,559*
Mn			x

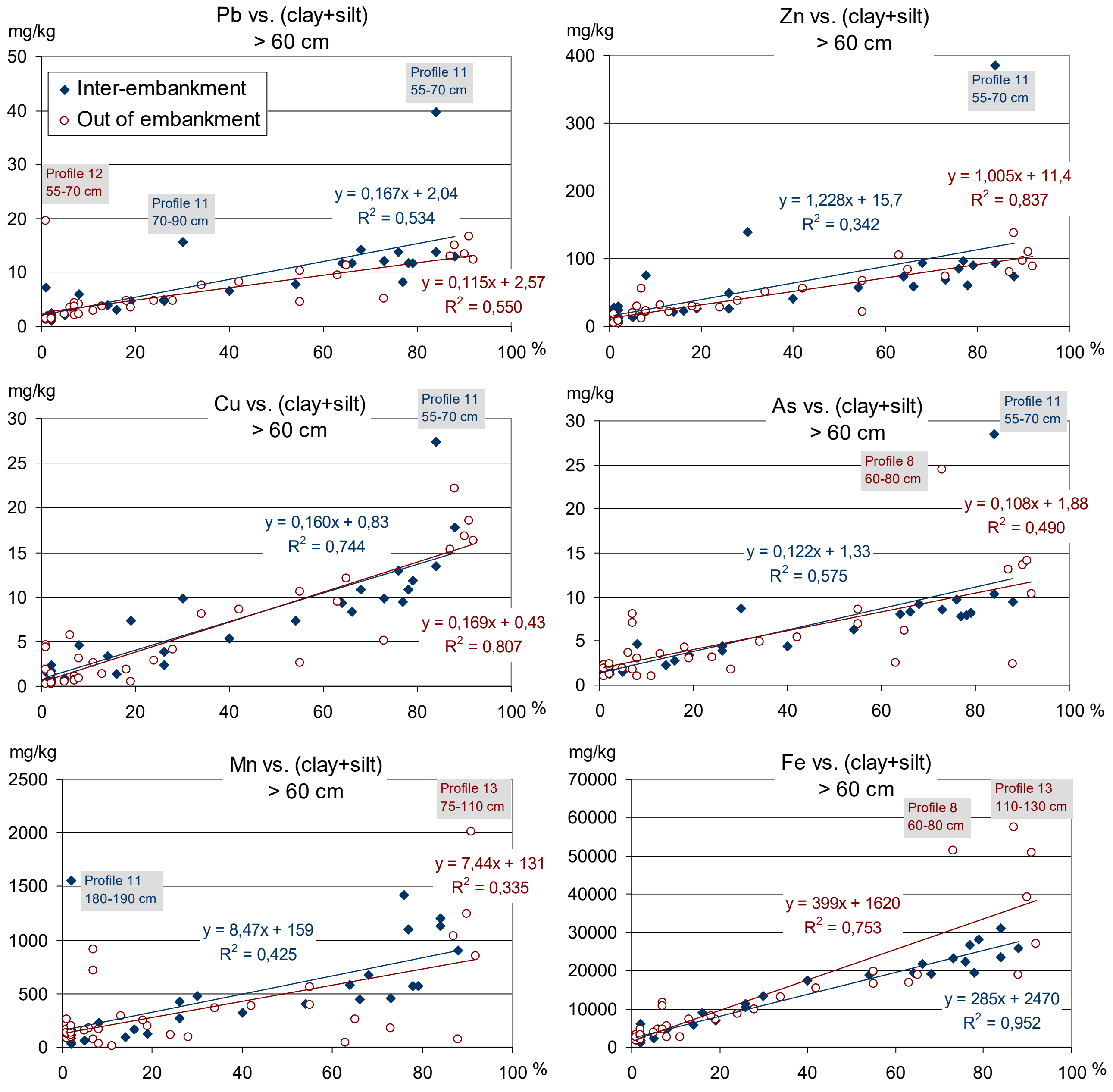

Figure S1. The relationships between soil concentrations of metal(loid)s and the sum of clay+silt fraction in the subsoil (>60 cm).
